# HUBS OF INNOVATION: GWEP UTILIZATION OF TECHNOLOGY AND INNOVATION FOR TRAINING AND SERVICE DELIVERY

**DOI:** 10.1093/geroni/igz038.1364

**Published:** 2019-11-08

**Authors:** Thomas V Caprio, Katherine A Bennett, Greg O’Barr, Catherine P Carrico, Christine McKibbin

**Affiliations:** 1 University of Rochester, Rochester, New York, United States; 2 University of Washington, Seattle, Washington, United States; 3 Cheyenne Regional Medical Center, Cheyenne, Wyoming, United States; 4 University of Wyoming, Laramie, Wyoming, United States

## Abstract

Technology offers powerful tools for meeting the increasing needs of older adults and caregivers. The Geriatric Workforce Enhancement Program recognizes the important role technology will play in service delivery and training by fostering innovation to meet local training and healthcare needs. This symposium will review the innovative models and strategies GWEPs are utilizing to improve care including Project ECHO, remote monitoring, and telehealth. The policy implications of these models will be presented, including current legislative action to support technology and innovation in the care of older adults.

